# Production of Injectable Marine Collagen-Based Hydrogel for the Maintenance of Differentiated Chondrocytes in Tissue Engineering Applications

**DOI:** 10.3390/ijms21165798

**Published:** 2020-08-12

**Authors:** Salvatrice Rigogliuso, Monica Salamone, Enza Barbarino, Maria Barbarino, Aldo Nicosia, Giulio Ghersi

**Affiliations:** 1 Abiel s.r.l, c/o University of Palermo, Viale delle Scienze, Ed. 16, 90128 Palermo, Italy; salvatrice.rigogliuso@unipa.it (S.R.); m.salamone@abielbiotech.com (M.S.); 2 Department of Biological, Chemical and Pharmaceutical Sciences and Technologies (STEBICEF), University of Palermo, Viale delle Scienze, Ed. 16, 90128 Palermo, Italy; enza.barbarino4@gmail.com (E.B.); mary.barbarino26@gmail.com (M.B.); 3 Institute for Biomedical Research and Innovation-National Research Council (IRIB-CNR), Via Ugo La Malfa 153, 90146 Palermo, Italy

**Keywords:** jellyfish collagen, composite injectable hydrogel, enzymatic cross-linking, cartilage, chondrocytes, differentiation, gene expression

## Abstract

Cartilage is an avascular tissue with limited ability of self-repair. The use of autologous chondrocyte transplants represent an effective strategy for cell regeneration; however, preserving the differentiated state, which ensures the ability to regenerate damaged cartilage, represents the main challenge during *in vitro* culturing. For this purpose, we produced an injectable marine collagen-based hydrogel, by mixing native collagen from the jellyfish *Rhizostoma pulmo* with hydroxy-phenyl-propionic acid (HPA)-functionalized marine gelatin. This biocompatible hydrogel formulation, due to the ability of enzymatically reticulate using horseradish peroxidase (HPR) and H_2_O_2_, gives the possibility of trap cells inside, in the absence of cytotoxic effects, during the cross-linking process. Moreover, it enables the modulation of the hydrogel stiffness merely varying the concentration of H_2_O_2_ without changes in the concentration of polymer precursors. The maintenance of differentiated chondrocytes in culture was then evaluated via morphological analysis of cell phenotype, GAG production and cytoskeleton organization. Additionally, gene expression profiling of differentiation/dedifferentiation markers provided evidence for the promotion of the chondrogenic gene expression program. This, combined with the biochemical properties of marine collagen, represents a promising strategy for maintaining *in vitro* the cellular phenotype in the aim of the use of autologous chondrocytes in regenerative medicine practices.

## 1. Introduction

It is known that cartilage self-repair is hampered by several factors mainly related to the limited tissue vascularization which results in a poor replicative potential of chondrocytes, as well the reduced chondrocytes propensity to migrate into the damaged site [1,2]. However, chondrocytes are recognized to be among the most versatile cell types due to their renewal/differentiation ability, which makes them suitable for various applications [3]. Regenerative medicine techniques based on the use of autologous chondrocytes transplantations via tissue engineering approaches provide promising evidence for the effectiveness of cell-based regeneration strategies, which lead to the regeneration of the damaged cartilage. Adult nasal chondrocytes (NC) are considered particularly worthy of attention due to their unique features [4]. They derive from the neural crest and have been recently shown to be able to respond and adapt to heterotopic transplantation sites. In particular, chondrocytes from the nasal septum allowed enhanced reproducibility in generating hyaline-like cartilage tissues, with superior plasticity to adapt to a joint environment, resulting in improved performances in tissue regeneration and repair [5,6]. *In vitro* isolation and expansion of NC are required to obtain an adequate quantity of cells. However, preserving the differentiated state, which ensures the ability to regenerate damaged cartilage, represents the main challenge during *in vitro* culturing. Therefore, several efforts are usually made to avoid dedifferentiation into fibroblast-like cells occurring via the reduction in collagen type II production and increased deposition of collagen type I. They include the addition of growth and differentiation factors, such as TGF-β [7] and the development of substrates as three-dimensional (3D) scaffolds that mimic the physiological environment ensuring adequate mechanical stimulation [8]. A lot of synthetic polymers are used to produce three-dimensional and highly porous scaffolds [9,10]; however, these polymers often require structural modifications or surface functionalization which increase their biocompatibility. To do this, it is possible to combine collagen with other bioactive molecules to obtain various modifications of the original polymer [11,12]. Nevertheless, hydrogels containing highly hydrated 3D networks mimicking the extracellular matrix (ECM) are highly recommended because of the similarity to native cartilage [13]. There is extensive evidence on the key role of the ECM, of which collagen type I is among the main components, to tune morphogenesis, development, cell growth, migration and tumorigenesis [14,15]. To date, natural biomaterials, including agarose, alginate and Matrigel [13], are often used for 3D scaffolding systems in biomedical applications. The combination of natural and synthetic polymers has been proven to provide greater mechanical stability and improve biocompatibility, acquiring from the synergistic properties of both materials. Interestingly, various combinations of collagen with other natural polymers such as inulin [16], or combination of alginate, gelatin and fibrin [17] have been transformed into formulations of injectable hydrogel enabling in situ formation [18]. Due to their ease of encapsulation of cells/biomolecules and subsequent administration in the absence of significant toxic effects, the use of injectable hydrogels represents an interesting strategy for tissue engineering and cell therapy [19]. This technology also enables the use of combined hydrogels as a drug release system thus replacing synthetic polymers such as poly(N-vinyl pyrrolidone) (PVP), currently used for this purpose [20,21,22]. The excellent biocompatibility and safety due to its biological characteristics, such as biodegradability and weak antigenicity, made collagen a preferred resource in biotechnological applications [23] both for the tissue engineering [8] and cosmetic and pharmaceutical industry [24].

Although collagen and ECM remodeling repertoire appeared within cnidarians in the first phase of evolution [25,26], the main sources for industrial and biomedical applications are from mammals including bovine and porcine [27]. However, several limitations occur in the use of these collagens. They include allergic responses, the possibility of transmitting zoonosis to humans as bovine spongiform encephalopathy (BSE) and foot-and-mouth disease (FMD) [28] and also restriction based on religion [29]. To overcome these issues nonmammalian collagen sources have also been evaluated. Among them, marine invertebrates as jellyfish possess high protein content, especially in collagen that accounts for up to 40–60% of dry weight [30]. In particular, collagen derived from *Rhizostoma pulmo* has been shown to have a high degree of similarity to mammalian type I collagen [31] and it has been also referenced to possess collagen type II-“like” properties [32,33]. Jellyfish collagen (JFC) shares several characteristics with the vertebrate counterpart including biocompatibility [31] and the possibility of supporting 2D and 3D cell cultures. Hence, it has been used for the production of microparticles enabling the controlled release of therapeutic proteins [34] and 3D highly porous scaffolds [35,36,37] or sponges [38] also supporting the chondrogenic phenotype and the redifferentiation of cells previously grown in a monolayer [33], thus enabling its application in cartilage tissue engineering [39]. Unfortunately, procedures allowing the polymerization of marine collagen in sponges or microparticles generally rely on a chemical cross-linking [27,32], which is incompatible with the simultaneous mixing of the cells in the solution, due to the potentially toxic residues remaining trapped within the material. Indeed, despite the biocompatibility of the material, these 3D supports require abundant washes, which allow the elimination of potentially toxic residues, before they are able to come into contact with the cells. Generally, cells are seeded on their surface and from there they can migrate inside until they colonize the entire volume of the matrix [8].

In order to overcome this limitation, herein we used *R. pulmo* collagen for the production of a highly biocompatible composite and injectable hydrogel, capable of enzymatic cross-linking, through oxidative coupling catalyzed by hydrogen peroxide (H_2_O_2_) and horseradish peroxidase (HRP). This formulation enabled the tuning of the hydrogel stiffness merely varying the concentration of H_2_O_2_ without changes in the concentration of the polymer precursor. Moreover, it allowed the trapping of cells inside it during the cross-linking process, in the absence of any cytotoxic effects. The efficiency of the described hydrogel in terms of biocompatibility and in the maintenance of differentiated state was evaluated morphologically, phenotypically and for the transcriptional expression of chondrogenic markers. The absence of negligible effects together with the injectability of the composite hydrogel suggests the possibility of its use in a preclinical step and cartilage treatment in clinical applications.

## 2. Results and Discussion

### 2.1. Purification and Characterization of R. pulmo Native Collagen

Natural biomaterial represents an interesting tool for the production of highly biocompatible injectable hydrogels. Because limitations in the use of mammalian collagen are well known, alternative sources of safe collagen have been sought especially in the marine environment. Marine collagen (MC) extraction can be usually carried out both in acidic and basic solutions or by enzymatic digestion, mainly through the use of pepsin [27,38].

**Figure 1:**
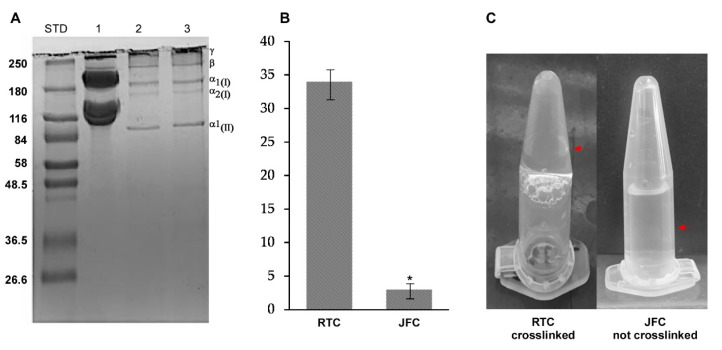
Acid-soluble proteins extracted from *R. pulmo* and comparison of the mammalian collagen from rat tail (RTC) and jellyfish collagen from *R. pulmo* (jellyfish collagen (JFC)). (**A**) SDS-PAGE analyses of rat tail type I collagen (20 μg; Line 1); *R. pulmo* oral arms collagen (20 µg; Line 2); *R. pulmo* umbrella collagen (20 µg; Line 3). STD represents molecular weight markers in kDa. (**B**) Analysis of cross-linking degree. Bars represent mean ± SD (*n* = 3), and asterisk denotes *p* < 0.05. (**C**) JFC was not able to gelify, unlike the RTC which forms a very stable hydrogel in a few minutes. The arrows indicate the different positions of the collagen in each sample.

However, to obtain jellyfish collagen in native form, we herein optimized the extraction of acid-soluble collagen avoiding pepsin digestion step. The *R. pulmo* acid-soluble proteins were purified as described in the experimental section and characterized by SDS-PAGE (Figure 1A).

Under reducing conditions, the acid-soluble proteins showed a pattern mainly consisting in α1 and α2 chains (approximately 180 kDa), and the β and γ chains located on a high molecular mass region (above 240 kDa), with a pattern likely resembling type I and type II collagen. This results in accordance with data on collagen isolated from several marine sources [36,38]. Additionally, the highly resolved protein pattern indicates that the used protocol allows the extraction of jellyfish collagen in native form. Although it is usually indicated a high correspondence between the collagen of mammalian and marine species [36], the apparent molecular weight of *R. pulmo* α and β chains was higher than the vertebrate counterparts. This could indicate variations in protein length and structural differences, probably due to their different amino acid composition. In this line, it has been observed that *R. pulmo* collagen contains less hydroxyproline, proline, glycine and glutamic acid residues than rat and bovine type I collagen [36]. Whilst these amino acids are involved in the formation and stability of the quaternary structure (triple helix) and thermal stability of type I collagen, the reduced content of these amino acids did not appear to affect the structure of *R. pulmo* collagen [40].

However, the analysis of cross-linking degree (Figure 1B,C) showed that a simple variation of pH, which is known to induce mammalian collagen polymerization [41], does not allow a stable gel formation on JFC at 37 °C.

Thus, it is reasonable to suppose that these residues on *R. pulmo* collagen are probably not available for cross-linking due to the lower percentage, or because they are structurally located in not accessible sites. This is consistent with the lower melting temperature (28.9 °C) which has been measured for *R. pulmo* collagen [31].

### 2.2. Functionalization Strategy for a Stable and Injectable Marine Hydrogel Formulation

**Figure 2:**
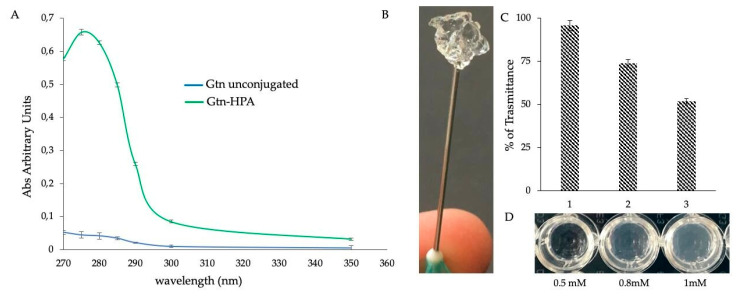
Functionalization of fish gelatin. (**A**) Analysis of the absorption spectra of fish gelatin (Gtn) after conjugation with hydroxy-phenyl-propionic acid (HPA; green curve), compared to the unconjugated Gtn used as a control (blue curve). Bars represent mean ± SD (*n* = 3). (**B**) Hydrogel of functionalized Gtn-HPA cross-linked by HPR and H_2_O_2_ enzymatic reaction. (**C**) Determination of hydrogel stiffness degree by transmittance analysis. The higher density of the gel, due to a higher cross-linking number, is inversely proportional to the percentage (%) of transmittance. The solution of Gtn-HPA uncross-linked was used as control. Transmittance was read at 700 nm. In correspondence with the histogram, the relative images of the hydrogel. Bars represent mean ± SD (*n* = 3). (**D**) show the increase in H_2_O_2_ concentrations, from 0.5 to 1 mM, augmented by the number of cross-linking and the opacity of the gel.

**Figure 3:**
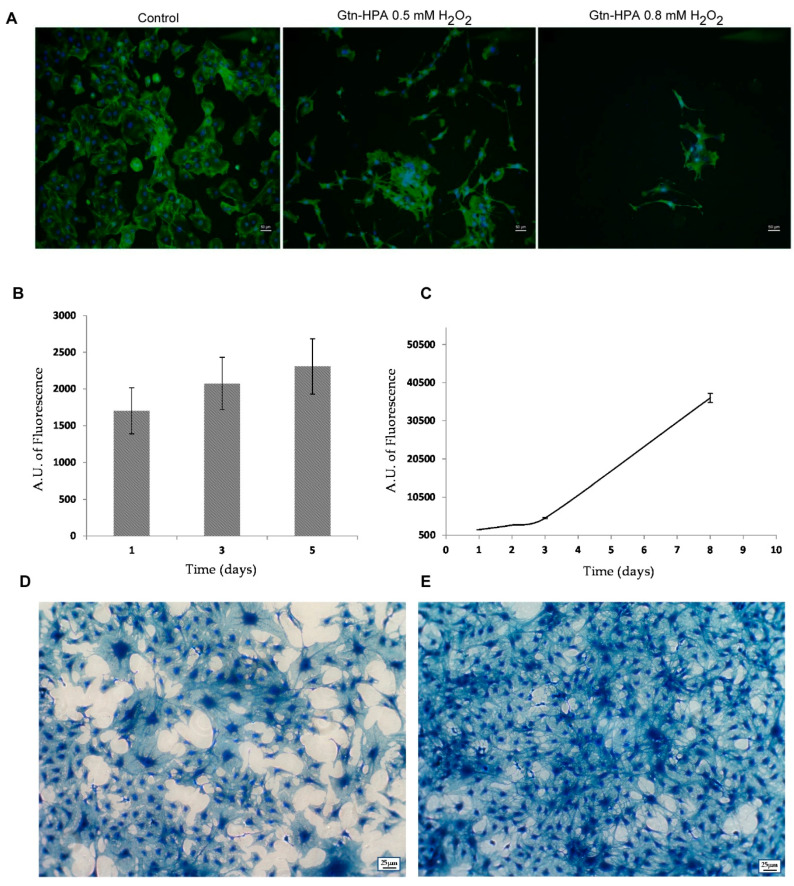
The use of 0.5 mM H_2_O_2_ for enzymatic cross-linking does not impair cell viability. (**A**) Different and nontoxic H_2_O_2_ concentrations do not modify cell morphology. MC3T3-E1 cells were grown, respectively, on cover glass as 2D control, in the Gtn-HPA hydrogel cross-linked with 2 U/mL HPR and 0.5 mM H_2_O_2_ and in the Gtn-HPA hydrogel cross-linked with 2 U/mL HPR and 0.8 mM H_2_O_2_ for 5 days. Cell cytoskeleton was stained with Phalloidin FITC (Green). Nuclei were stained with DAPI (Blue). Magnification was 10×. Scale bar was 50 µm. (**B**) Proliferation assay of MC3T3-E1 cell growth within the Gtn-HPA hydrogel cross-linked with HPR and 0.5 mM H_2_O_2_. Bars represent mean ± SD (*n* = 3) (**C**) proliferation assay of MC3T3-E1 cells recovered after 5 days in the Gtn-HPA hydrogel and then seeded on 2D; (**D**) contrast microscopy images of MC3T3-E1 cells after hydrogel recovery and seeded on 2D for 3 days and (**E**) for 8 days. Cells have been stained with Coomassie Brilliant Blue G. The magnification was 5×; scale bar was 25 μm.

In order to overcome limitations in the use of native *R. pulmo* collagen on the formation of a stable hydrogel, denatured marine collagen (gelatin) was functionalized and used for the creation of a combined marine hydrogel. Enzymatic cross-linking agents are recognized to promote gel formation [42] eliminating the risk of cytotoxic effects. Based on this, we functionalized fish gelatin, through the binding of hydroxy-phenyl-propionic acid (HPA) from its reactive group COOH, making gelatin rich in potentially reactive OH groups (Figure 2A). These groups, in the presence of H_2_O_2_ and horseradish peroxidase (HRP), are known to react so as to obtain a hydrogel [43] with a stable cross-linking (Figure 2B) and increasing stiffness. This depends on the concentration of used H_2_O_2_, (from 0.5 mM to 1 mM) as indicated by transmittance analysis (Figure 2C) and, as well, increase of opacity of each hydrogel (Figure 3D). As regards the gelation time, it resulted independently of the concentration of H_2_O_2_ herein used, as the hydrogel always forms in less than 2 min at 37 °C. Moreover, a further increase in the H_2_O_2_ amount failed in gel formation probably due to the inhibition of HRP for an H_2_O_2_ excess [43].

In order to obtain high gelation yield, alongside with the absence of harmful effects on cells, it is mandatory the identification of a proper ratio between HPR and H_2_O_2_. Therefore, to establish that MC3T3-E1 cell culture, which is known to be impaired by H_2_O_2_ exposure [44], was mixed with Gtn-HPA separately at 0.5 and 0.8 mM H_2_O_2_. Hydrogels with increasing density were obtained and the cells were monitored for five days. Microscopic analysis revealed that both tested H_2_O_2_ concentrations do not modify cell morphology (Figure 3A) compared to MC3T3-E1 grown in monolayer and herein used as control (Figure 3A). Interestingly, the different gel stiffnesses appeared to influence cell proliferation because an evident reduction occurred at 0.8 mM H_2_O_2_ (Figure 3A).

However, as measured in cell proliferation assays (Figure 3B), both the cells inside the hydrogel and those recovered from this and seeded in 2D (Figure 3C), showed a linear proliferation rate over time, although slower in 3D than in 2D. The cell’s ability to continue to proliferate once extracted from the gel was also monitored for eight days (Figure 3C–E) indicating the absence of any cytotoxic effect experienced during the enzymatic polymerization.

**Figure 4:**
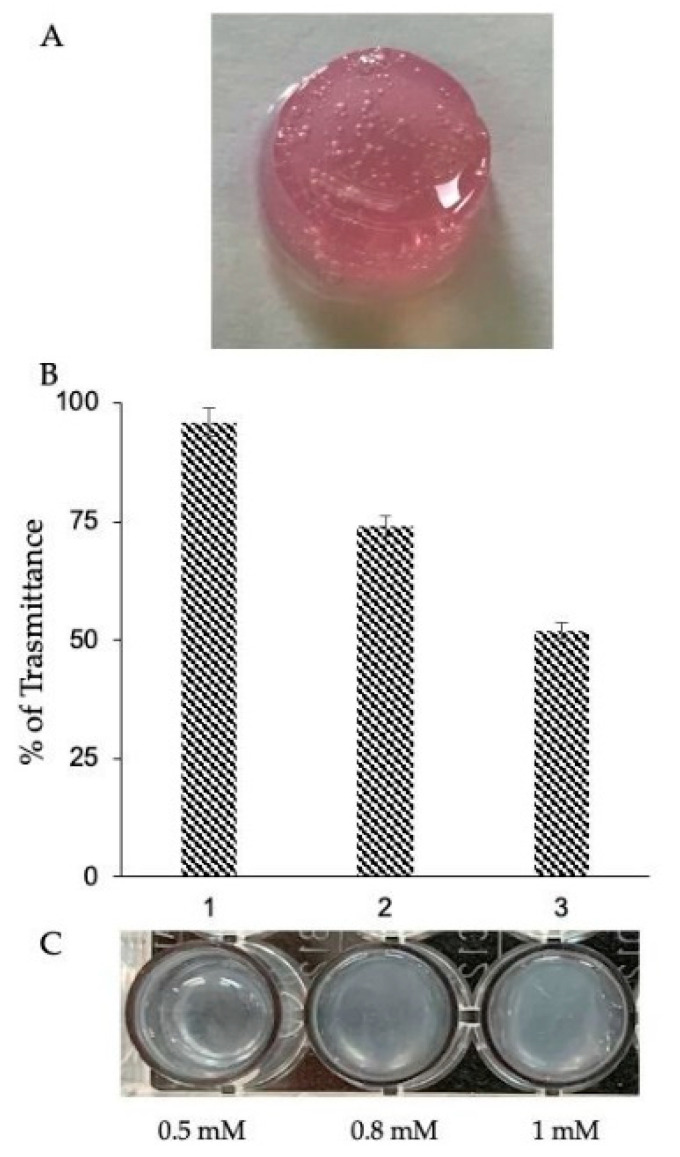
Stable and injectable combined marine hydrogel. (**A**) Production of injectable hydrogel totally from marine source (Marine Collagen hydrogel (MCh)) by mixing (1:1) JFC in native form and functionalized denatured collagen (Gtn-HPA) obtained by the reaction of HPR 2 U/mL and 0.5 mM H_2_O_2_. The use of the lower concentration of H_2_O_2_ minimizes the possible toxicity of the system. The hydrogel formulation has shown perfect flow from 23 G and thicker-sized syringes. (**B**) Determination of hydrogel stiffness degree by transmittance analysis. The higher density of the gel, due to a higher cross-linking number, is inversely proportional to the percentage (%) of transmittance. The solution of MC uncross-linked was used as control. Transmittance was read at 700 nm. Bars represent mean ± SD (*n* = 3). In correspondence with the histogram, the relative images of the hydrogel (**C**) show how the increase in H_2_O_2_ concentrations, from 0.5 to 1 mM, augmented the number of cross-linking and the opacity of the gel.

In order to obtain a fully marine and stable 3D hydrogel (Marine Collagen hydrogel (MCh)), we created an injectable collagen-based hydrogel, by mixing in equal proportions the *R. pulmo* native collagen and the Gtn-HPA (Figure 4A). This formulation enables the possibility of tuning the stiffness of the hydrogels varying the concentration of H_2_O_2_ (Figure 4B,C) without changing the concentration of the polymer precursor. Moreover, it allows the trapping of cells inside the gel during the cross-linking process, in the absence of cytotoxic effects. In addition to the advantages related to the used biomaterial [33], such combined formulation also exhibits a sol–gel phase transition which enables injection and in situ gelation through a 23 G needle with a perfect flow.

### 2.3. Comparing Rat Tail Collagen Hydrogel (RTCh) and MCh for the Maintenance of Differentiated Chondrocytes

Many pieces of evidence support the biochemical properties of marine collagen on chondrocyte differentiation [33,39,45], thus suggesting its use in cartilage engineering and tissue repair.

The effectiveness of MCh in the maintenance of a differentiated chondrocyte phenotype was then evaluated and compared with a canonical hydrogel of type I collagen (Rat Tail Collagen hydrogel (RTCh)). NCs were seeded, respectively, within the RTCh and MCh and their behavior was monitored over time. It is known that NCs possess the ability to remodel RTCh [46].

**Figure 5:**
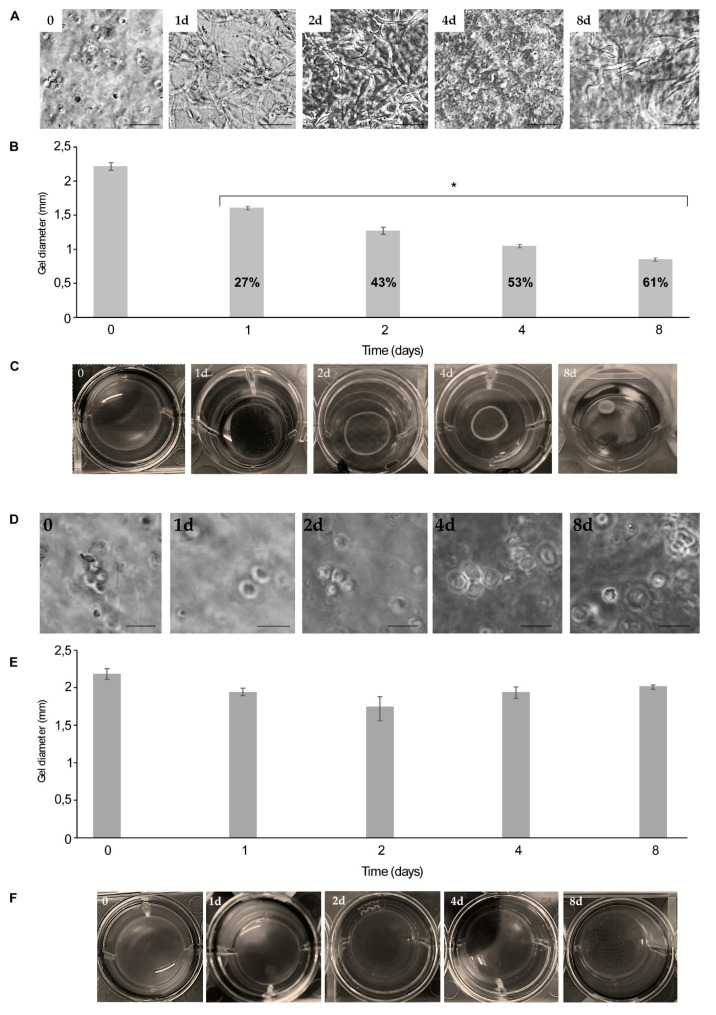
Performance of Rat Tail Collagen hydrogel (RTCh) and MCh in the maintenance of chondrocytes. (**A**) Growth of bovine nasal chondrocytes (NCs), time 0 control (0) and growth within RTCh, respectively, for 1 (1d), 2 (2d), 4 (4d) and 8 days (8d). Magnification was 20×; scale bar was 25 μm. NCs remodeled RTCh (type I collagen) from the first day of culture; this was indicated both by the histogram of the reduction of the RTCh diameter over time (**B**), in which 61% of contraction with respect to time 0 was observed, after 8 days of culture, and from direct images of the hydrogel (**C**) that show how the NCs growth rate was responsible for the progressive and evident remodeling of the hydrogel. (**D**) Growth of bovine nasal chondrocytes (NCs), time 0 control (0) and growth within MCh, respectively, for 1 (1d), 2 (2d), 4 (4d) and 8 days (8d). Magnification was 20×; scale bar was 25 µm. (**E**) NCs do not significantly remodel the MCh. After the first day of culture about 7% of the gel contraction was observed; the same value has been maintained over time. (**F**) Images of MCh during the NCs culturing, no evident reduction in gel diameter occurred. Bars represent mean ± SD (*n* = 3) and * denotes *p* < 0.05.

As shown in Figure 5, the fast NC proliferation rate (Figure 5A) was responsible for the progressive contraction of the RTCh (Figure 5B,C). In particular, herein we reported that the process begins at Day 1 (Figure 5C) and measurements in the variation of the RTCh diameter indicated a gradual gel contraction that reaches 61% after eight days (Figure 5B).

Differently, the NCs within MCh (Figure 5D) do not possess the same remodeling ability; the measurements of the variation of the MCh dimensions showed that the 3D matrix diameter was maintained over time (Figure 5E,F). Previous data indicated that the inability to contract the gel is closely related to the maintenance of the chondrogenic phenotype [46]. Interestingly, NCs grown in RTCh showed a high proliferation rate associated with a transition from a rounded to a spread morphology (Figure 5A). Conversely, NCs maintained a rounded morphology over time in MCh (Figure 5E).

**Figure 6:**
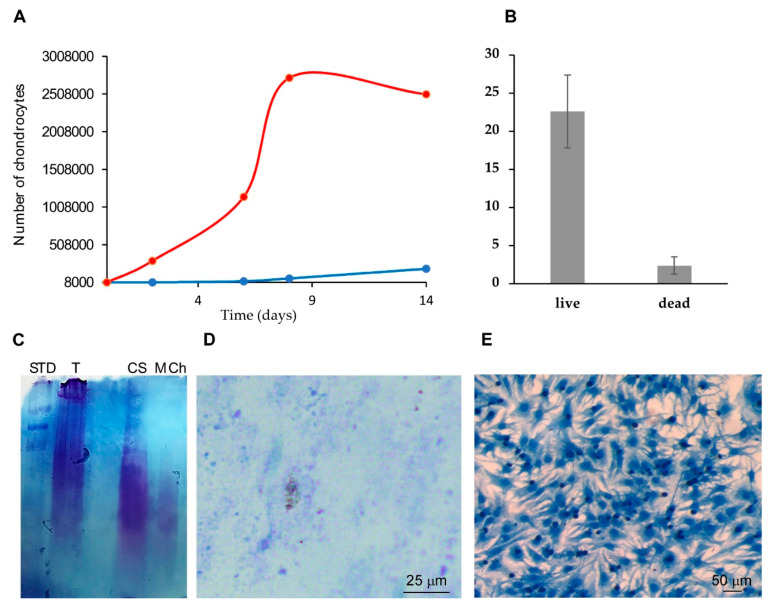
Chondrocytes in MCh are viable and produce glycosaminoglycan (GAG). (**A**) Proliferation assay of chondrocytes in 3D within MCh (blue curve) and in monolayer (red curve). (**B**) Viability evaluation by Trypan Blue staining the cells grown within the MCh for 14 days. NCs showed 90% of viability Bars represent mean ± SD (*n* = 3). (**C**) Electrophoretic analysis of the matrix extracted from MCh after 14 days of NCs culturing. SDS-PAGE were stained with Alcian Blue (STD: molecular weight marker); T: Digested Bovine cartilage; CS: Chondroitin Sulfate control; MCh: Digested MCh after 14 days of NCs culturing. (**D**) Light micrograph of NCs growth within MCh for 14 days, stained with Alcian Blue. Magnification was 20×. Scale bar was 25 µm. (**E**) Chondrocytes resumed after hydrogel digestion and seeded in 2D conditions on a plate. Cell proliferation was very slow inside MCh; after, seeding cells started to proliferate and reached confluence after a few days.

Additionally, a reduced cell proliferation rate compared to NCs grown in 2D, herein used as a control, was measured in MCh (Figure 6A).

Given the low proliferative rate within the MCh, we wondered to evaluate the degree of cell viability. As indicated in Figure 6B the cells grown in MCh for 14 days showed 90% of viability thus ensuring that MCh does not exert any toxic effect.

To evaluate the use of such hydrogels for chondrogenic differentiation, the production of a matrix rich in glycosaminoglycan (GAG), as a proxy of chondrogenic activity [47], was analyzed. NCs grown in MCh were able to deposit the GAG-rich matrix as revealed via the Alcian blue staining of the electrophoretic pattern of GAGs extracted from MCh after 14 days of NCs culturing (Figure 6C). Similarly, the direct MCh staining (Figure 6D) also showed GAGs produced by the chondrocytes within the MCh. Thus, GAGs accumulated in the hydrogels, reflecting the feasibility of using MCh for active chondrocytes maintenance.

As further evidence of cell viability, the chondrocytes were recovered after hydrogel digestion by collagenases treatment and seeded in 2D conditions; the slow proliferation rate within MCh, changed after NCs seeding in monolayer. Cells began to proliferate with a physiological rate and reached confluence in a few days (Figure 6E).

Based on the round morphology (chondros) observed in MCh, the low degree of proliferation and the GAG production therein, it is likely to hypothesize the maintenance of the differentiated phenotype in MCh.

### 2.4. Cytoskeleton Organization and Gene Expression Analyses

**Figure 7:**
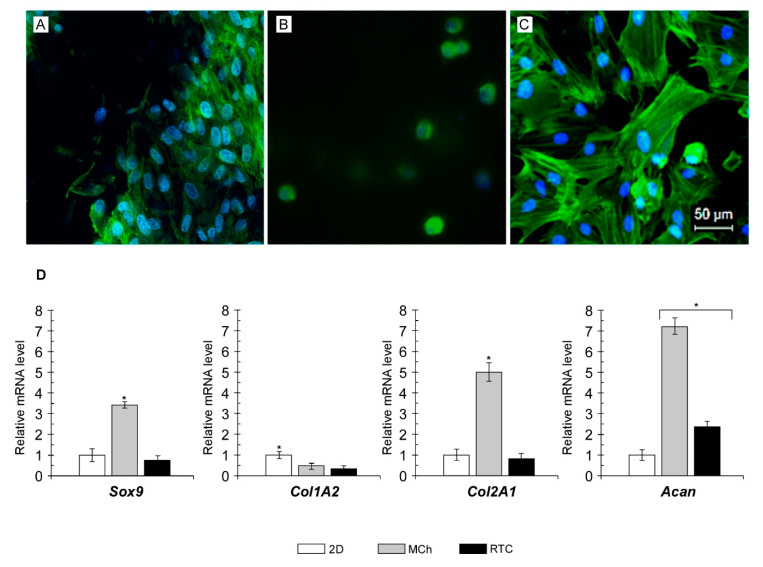
Culturing in MCh does not alter cytoskeleton organization and NCs express markers of the differentiated state. Staining of NCs after 8 days of culture, respectively, within RTCh (**A**), MCh (**B**) and 2D (**C**). It is possible to observe the characteristic cytoskeletal localization of F-actin in NCs grown under 2D conditions (**C**); a more widespread distribution has been observed in RTCh (**A**). The localization of F-actin is almost not observed in NCs grown in MCh (**B**), which maintains the morphology round over time. The F-actin cytoskeleton was stained with Phalloidin FITC (green); the nuclei were stained with DAPI (blue). Magnification was 10×; scale bar was 50 µm. (**D**) Gene expression analyses of *Sox9*, *Col2A1*, *Col1A2* and *Acan* by qRT-PCR. The gene expression levels were analyzed using the −ΔΔCt method using 18S rRNA and GAPDH as the internal controls. The data represent the mean ± SD of three independent culture experiments. Statistical analysis by one-way ANOVA. The symbol * indicates *p* < 0.05.

The dedifferentiation of chondrocytes is usually associated with changes in cell morphology, from rounded to spread. However, cell morphology is not always linked to phenotype. It has been shown that the loss of hallmarks for differentiated chondrocytes, including high expression of collagen type II, low expression of collagen type I [48] and a low proliferation rate [49], is also associated with an increase in the presence of F-actin cables within the cytoplasm of dedifferentiated cells [33,50]. In addition, because proteoglycan and collagen synthesis are correlated with the actin localization and cytoskeleton organization [50], efforts were made to examine the presence and the status of F-actin in the cytoskeleton of chondrocytes after culturing in 2D, MCh or RTCh. Therefore, cells were stained with phalloidin and the cytoskeletal organization was observed. As shown in Figure 7, the NCs staining after eight days of culturing in 2D showed the characteristic cytoskeletal localization of F-actin indicating the existence of conditions leading to dedifferentiation (Figure 7C). A more widespread distribution was observed in RTCh (Figure 7A). Evidence for an F-actin localization was negligible in NCs from MCh (Figure 7B), which once again maintained the round morphology over time.

Thus, it is reasonable to suppose that the chondrogenic phenotype was better maintained in MCh rather than the canonical 3D collagen type I hydrogel.

In order to confirm such assumption and evaluate the differentiated state after culturing in different conditions, the expression pattern of some cartilage-specific genes, including *Sox9*, *Col2A1* and aggrecan (*Acan*) as well as *Col1A2* as a proxy of fibrous differentiation [51] was evaluated and compared with that observed from cells grown in monolayer (Figure 7D)*. Sox9*, *Col2A1* and *Acan* were overexpressed (3.3-fold, 4.9-fold and 7.2-fold, respectively) in chondrocytes from MCh. The expression of a proxy of dedifferentiation, namely, *Col1A2*, was hugely reduced when compared with chondrocytes from 2D culture. Cultured cells from type I collagen hydrogel (RTCh) showed a different pattern of gene expression. No significant changes in the *Sox9* and *Col2A1* transcript levels occurred in such samples, which remained similar to those observed in control 2D cells. Interestingly, chondrocytes in RTC showed reduced *Col1A2* mRNA levels and increased *Acan* expression up to 2.3-fold then the control. It is recognized that the chondrogenic differentiation mainly relies on the activating role of Sox9 in the expression of chondrocyte-specific genes including *Col2A1* and *Acan* [52,53]. This suggests that MCh provides an adequate 3D environment supporting the activation of specific patterns of gene expression. These, in turn, act to maintain the chondrocyte-specific phenotype including the native spherical morphology as well the production of accumulation of proteoglycans and type II collagen. Although the use of type I collagen is common practice in cell culturing, especially for practical reasons, several pieces of evidence suggest the use of composite hydrogels including other compounds as type II collagen [49]. It has been shown that type II collagen provides a suitable environment for preserving the chondrocyte-specific phenotype [54,55]. The RT-qPCR analysis herein revealed that chondrocytes cultured in RTC do not express the gene characteristic of the dedifferentiated chondrocyte phenotype (*Col1A2*); however, the lack of *Sox9* and *Col2A1* overexpression appear to not fully sustain a chondrogenic gene expression pattern ensuring the maintenance of a differentiated state. It is noteworthy that the activity of Sox9 is also regulated by post-transcriptional modification and mechanical stimuli [56]. Additionally, it has been shown that *Acan* expression was achieved also after Sox9 knockdown [57]. Therefore, it is unsurprising that qPCR analyses showed significant *Acan* upregulation in the absence of increased *Sox9* expression as occurred in RTC 3D chondrocytes cultures.

## 3. Materials and Methods

### 3.1. R. pulmo Collagen Extraction

Two large jellyfishes, of the genus *R. pulmo*, were caught and immediately sectioned to separate the umbrella from the oral arms, the samples were placed in an ice bath until processing (about 2 h). Samples were kept in dH_2_O for 10 days in at 5 °C ensuring dH_2_O changes every 8 h.

From the 6th to 10th day, water conductivity was measured to monitor the amount of salt dissolved. Changes of clean dH_2_O water were done until complete desalination was obtained as compared to dH_2_O used as control. Acid-soluble proteins, mainly collagen, were extracted in the presence of acetic acid at a final concentration of (0.5 N; Tables S1 and S2, Supporting Information). The protein extraction process was carried out at 4 °C for a total of 20 days (Figure S1, Supporting Information) in the presence of increasing acetic acid amount as follows: Days 1–6 acetic acid at a final concentration of (0.05 N), Days 7–16 acetic acid at a final concentration of (0.1 N) and Days 17–20 acetic acid at a final concentration of (0.5 N). Then samples were passed by pipetting up and down through a 25 mL pipette in order to obtain a homogeneous solution, which was maintained in acetic acid (0.5 N) for a further 10 days under stirring, at 4 °C. Samples were then centrifuged at 10,000× *g* for 15 min at 7 °C; insoluble fraction was discarded; the collagen, acid-soluble protein, was collected and dialyzed in acetic acid solution, final concentration (0.02 N). The dialysis was carried out in 5 L of acid solution, in static conditions; the solution was changed 2 times a day, for a total of 5 days.

### 3.2. Characterization of R. pulmo Extracted Proteins

Samples obtained after the dialysis process were resuspended in Sample Buffer (Tris Base 2M pH 6.8; 2.5% SDS; 5% β-mercaptoethanol; 10% glycerol; 0.002% bromophenol blue) and heated to 100 °C for 3 min. Insoluble material was removed by centrifugation (2 min at 10,000× *g*). An amount of 20 µg of each sample was resolved on SDS-PAGE. The protein bands were visualized by Coomassie Blue staining (0.8% Coomassie Brilliant Blue G-250 in Methanol/distilled water/acetic acid 5:5:1). The protein’s molecular weight was estimated using a prestained molecular weight marker (Sigma Aldrich, Milan, Italy).

### 3.3. Natural Cross-Linking Degree Determination

The degree of natural *R*. *pulmo* collagen cross-linking was determined by ninhydrin assay which could measure the percentage of free amino groups remaining in the collagen before and after cross-linking [38]. In the ninhydrin assay, 1 mg/mL of collagen type I from rat tail (RTC; Corning, Turin, Italy), was used as control because of its ability to form a gel stable at 37 °C, pH 7.0. The same gelling protocol has been used for 1 mg/mL of jellyfish collagen (JFC). A 150 µL volume of each sample was mixed with 600 µL of ninhydrin solution and 30 µL TIN(II) chloride; the reaction was developed at 100 °C for 15 min in thermomixer at 20 g, then cooled in an ice-water bath. An equal volume (200 µL) of each solution was added into 1 mL 50% isopropanol and the optical absorbance at 570 nm (Abs 570) was measured by a spectrophotometer (SPECTROstar Nano, BMG Labtech, Ortenberg, Germany). The amount of free amino groups is related to the value of Abs 570; glycine at various known concentrations was used to create a standard curve (glycine concentration vs. absorbance). The degree of cross-linking of the sample was then calculated using the following equation:

(1) $$\frac{NH_{2}o-NH_{2}c}{NH_{2}o}\times 100$$

where *NH*_2_*o* is the free *NH*_2_ concentration in non-cross-linked samples and *NH*_2_*c* is the free *NH*_2_ concentration in cross-linked samples.

### 3.4. Synthesis of Gelatin (Gtn)-HPA Conjugate

3,4-hydroxyphenylpropionic acid (HPA) was used to synthesize Gtn–HPA conjugates by a general carbodiimide/active ester-mediated coupling reaction in MES buffer [43]. In this case, the carboxyl groups of HPA react with the amino groups present along the respective amino acid chain of Gtn. HPA (20 Mm) was dissolved in a mixture of MES buffer (0.5 M) pH 4.7 and *N*,*N*-dimethylformamide (DMF; 3:2). To this, 10 mM NHS (*N*-hydroxysuccinimide) and 20 mM EDC (1-ethyl-3-(3-dimethylaminopropyl)-carbodiimide hydrochloride) were added. The reaction was stirred at room temperature for 1 h at pH 4.7. Then, the same volume of Gtn solution 5% (*w*/*v*), dissolved in 0.5 M MES buffer pH 4.7 was added to the reaction mixture and stirred overnight at room temperature. The solution was transferred to dialysis tubes with a molecular cut-off of 14 kDa. The tubes were dialyzed against 100 mM sodium chloride solution for 2 changes and distilled water for a further 4 changes. The purified solution was preserved at 20 °C until use.

### 3.5. Enzyme-Mediated Injectable Hydrogels.

MC3T3-E1 (osteoblastic cell line from mouse calvaria; ECACC) culture was resuspended in Gtn-HPA solution. Then, HPR (2 U/mL) and 0.5 mM or 0.8 mM H_2_O_2_ were added. The samples were immediately incubated at 37 °C with 5% of CO_2_; the hydrogels were formed after a few minutes and fresh complete medium was added to each gel. Dulbecco’s modified Eagle’s medium (Sigma Aldrich, Milan, Italy) supplemented with 10% fetal calf serum (Sigma Aldrich, Milan, Italy), 1% glutamine (Euroclone, Milan, Italy), 1% antibiotics (streptomycin and penicillin; Euroclone, Milan, Italy) was used.

### 3.6. Marine Collagen Hydrogel (MCh) Preparation and Qualitative Evaluation of Injectability

JFC (5 mg/mL) and Gtn-HPA solutions were mixed (1:1) *v*/*v* and buffered with 1 N NaOH until pH 7. In order to restore a proper saline concentration in the sample, Hank’s salt solution was added to the marine collagen mix (MC) together with 2 U/mL HPR. Then the MC was split into 2 sterile tubes, respectively, containing 0.5 mM and 0.8 mM H_2_O_2_ and NCs were resuspended at a concentration of 5 × 10^5^ cells/gel. The solutions were gently mixed together and 500 µL/well were seeded in a 24 well plate. Samples were incubated at 37 °C with 5% of CO_2_ and MCh was formed in about 2 min; fresh complete medium was added to each well. Dulbecco’s Modified Eagle’s Medium/Nutrient Mixture F-12 Ham (Sigma Aldrich, Milan, Italy) supplemented with 10% fetal calf serum (Sigma Aldrich, Milan, Italy), 1% glutamine (Euroclone, Milan, Italy), 1% antibiotics (streptomycin and penicillin; Euroclone, Milan, Italy) was used for the maintenance of chondrocytes in culture. MCh was prepared as described above, then, HPR and H_2_O_2_ were added for testing injectability of the resulting hydrogel. The hydrogel formulation was injected in situ through a 23 G needle. The gelation occurs about after 2 min from the addition of H_2_O_2_.

### 3.7. Isolation of Nasal Septal Chondrocytes from Bovine Tissue

Chondrocytes from bovine nasal septa were isolated using recombinant collagenase as described elsewhere [51,58,59]. One gram of minced tissue was incubated with a mixture of 300 units of Collagenase H (Abiel, Palermo, Italy) plus Thermolysin (Promega, Milano, Italy) 250 µg in 10 mL of Dulbecco’s Modified Eagle’s medium (Sigma, Milan, Italy) containing 1% Pen–Strep and 1% Fungizone (Euroclone, Milan, Italy). The sample was digested for 18 h at 37 °C in 5% CO_2_.

After digestion, the collagenase/chips solution was filtered through a 20 μm nylon filter membrane.

After centrifugation for 10 min at 250 g, the pellet containing chondrocytes was washed twice in DMEM. The cells were then suspended by adding 20 mL of medium, and cell viability was assessed using Trypan Blue exclusion test. Cells were seeded at an initial density of 5 × 10^3^ cells/cm^2^ in T-175 flasks in DMEM supplemented with 10% fetal bovine serum (FBS Sigma, Milan, Italy) + 1% Pen–Strep + 1% Fungizone and 50 µg/mL acid ascorbic (Sigma, Milan, Italy). Cell cultures were expanded in an incubator humidified at atmospheric pressure at 37 °C and 5% CO_2_.

### 3.8. Immunofluorescence Assay

MC3T3-E1 cells grown in 2D conditions on the cover glass and NCs 3D cultures within RTCh and MCh, were fixed with 3.7% paraformaldehyde for 12 min. Subsequently, the samples were washed thoroughly with phosphate buffer saline (PBS 1×). Cells were stained with DAPI (1:20,000 Sigma, Milan, Italy; blue nuclei) and Phalloidin FITC (1:500 Sigma, Milan, Italy; green cytoskeleton) for 20 min at 37 °C. The cover glass was mounted with a mounting solution (glycerol 80%; NaN_3_ 0.01% on 0.2 M Tris HCl pH 7.5). The samples were analyzed by an epifluorescence microscope (Leica, Mannheim, Germany).

### 3.9. Cell Viability and Proliferation Assays

Cells from 3D hydrogels (Gtn-HPA or MCh) were recovered via hydrogels digestion in the presence of 1 mg/mL collagenase G/H mix (1:10; Abiel, Palermo, Italy) for 60 min at 37 °C. Viability assay from 3D hydrogel or from 2D condition cells was assessed using Trypan Blue exclusion test. The cells were washed with PBS and counted in the Burker chamber. The cells recovered after gel digestion were seeded on a plate and their proliferative capability was monitored over time using the Alamar-Blue colorimetric assay (Thermo Scientific, Foster City, CA, USA) according to the manufacturer’s recommendations. Cells were incubated at different times, with an Alamar-Blue reagent solution (1:10) in their complete medium for 2 h in a humidified incubator (37 °C; 5% CO_2_). The increase in fluorescence was evaluated by a microplate reader (Synergy HT, Biotek, Winooski, VT, USA) using excitation/emission wavelengths of 530/590 nm. The standard curve was obtained through the use of known cell concentrations. Cells were stained with Coomassie Brilliant Blue G (0.8% Coomassie Brilliant Blue G-250 in Methanol/distilled water/acetic acid 5:5:1) for 10 min, then their morphology was assessed by phase-contrast microscopy (Leica).

### 3.10. RTCh Preparation

3D RTC hydrogels were produced from high concentration type I collagen (BD Biosciences, San Jose, CA, USA), using the protocol described from Salamone et al, 2010 with some modifications [41]. Type I collagen was diluted to a concentration of 1 mg/mL with Hank’s salt solution 1×, then it was buffered with NaOH 1 N until pH 7.0. NCs (5 × 10^5^), suspended in growth media and added to the collagen solution. Then, 500 µL/well of RTC solution was immediately seeded into 3 wells of a 24-well plate and the samples were incubated at 37 °C with 5% CO_2_. Cells grown in the 3D matrices were visualized using both a phase-contrast microscopy and epifluorescence microscope (Leica).

### 3.11. Gel Contraction Assay

Nasal chondrocytes (5 × 10^5^/gel) seeded, respectively, within RTCh and MCh were placed in 24-well plates and incubated in the chondrocyte maintenance medium for 1, 2, 4 and 8 days. At different culture times, the diameter of each gel was evaluated with a ruler. The extent of contraction of the gels was expressed as the percentage of the initial area. Each experiment was performed in triplicate.

### 3.12. Glycosaminoglycans Identification of Electrophoretic Gel

The polyacrylamide gel electrophoretic technique (SDS-PAGE 10%) was used to identify and analyze the glycosaminoglycans (GAG) [60] produced by NCs grown in MCh. The MCh samples were dissolved using a mixture of 1 mg/mL G/H collagenase with a ratio of (1:10); samples were incubated for 60 min at 37 °C. After centrifugation at 15,000× *g* for 10 min, the supernatant was recovered, mixed with sample buffer (Tris Base 2M pH 6.8; 2.5% SDS; 5% β-mercaptoethanol; 10% glycerol; 0.002% bromophenol blue) and heated for 10 min; then 25 μL of each sample was loaded into the gel wells. Digested cartilage and purified chondroitin sulfate (CS) were used as a control. The electrophoretic gel was stained with Alcian Blue for 30 min at room temperature under gentle stirring (0.1% Alcian Blue on Ethanol/CH_3_COOH: (60%/40%)). Alcian Blue staining was also used to identify the GAG-rich matrix directly in MCh with NCs in culture for 14 days.

### 3.13. Statistical Analysis

All the experiments were performed in triplicate and data were expressed as mean ± SD. The differences between different samples were evaluated using a one-way ANOVA test and *p* < 0.05 was considered of statistical significance.

### 3.14. RNA Isolation and cDNA Synthesis

Total RNA was extracted using Trizol reagent (Invitrogen, CA, USA) according to the manufacturer’s instructions from 10 d chondrocytes cultured in the 2D system, in 3D collagen type I hydrogel and in the composite marine hydrogel. RNA concentrations and quality were verified by spectrophotometry via optical density (OD) at 260 nm, while the RNA integrity was checked on 1.5% agarose gel. The RNA was stored at −80 °C for future use. The extracted RNA (1 μg) was treated with RNA qualified 1 (RQ1) RNase-Free DNase (Promega, Madison, WI, USA) to remove any residual genomic DNA contamination, and the DNase was inactivated by adding 25 mM EDTA. First-strand cDNA was synthesized from 500 ng DNase-treated total RNA samples using random primers and High Capacity cDNA Reverse Transcription Kit (Life Technologies Corporation, Carlsbad, CA, USA), following the manufacturer’s instructions. The cDNA mixtures were stored at −20 °C.

### 3.15. RT-qPCR

RT-qPCR was performed using the BIO-RAD CFX96 System with Power Sybr Green as detection chemistry (Applied Biosystems, Foster City, CA, USA). Real-time PCRs were carried out in 20 µL mixture containing 1 µL of a 1:10 dilution of the cDNA preparations, using the following PCR parameters: 95 °C for 10 min, followed by 40 cycles of 95 °C for 10 s, and 60 °C for 60 s. Primer pairs used for qPCR are reported in Table 1. Amplifications were run in triplicate. The absence of nonspecific products was confirmed by both melting curves analysis and electrophoresis in 2% agarose gels. The 18S rRNA, and GAPDH were chosen as reference genes. A normalization factor was calculated based on geometric averaging of the expression level of these reference genes and was used to quantify the expression levels of the target genes using the −ΔΔCt method. All data represented relative mRNA expressed as the mean ± S.D. (*n* = 3). Significant differences between the values of the different treated groups and the reference control groups were determined by one-way ANOVA using Statistica 6.0 (StatSoft, Tulsa, OK, USA).

## 4. Conclusions

Herein we report the formulation of a collagen-based marine hydrogel able to cross-link with the cells trapped inside, by in situ injection in the presence of H_2_O_2_ and HPR, without any cytotoxic effect. The round morphology associated with the absence of a marked localization of cytoskeletal F-actin, the low degree of proliferation within the MCh and the inability of the NCs to contract the MCh, GAG deposition and the transcriptional expression of chondrogenic markers (namely *Sox9*, *Col2A1* and *Acan*) strongly suggest the maintenance of the chondrogenic phenotype.

All these features lead us to suppose that MCh has a greater potential to maintain the chondrocyte phenotype, compared to other 3D collagen hydrogels. Because chondrocyte dedifferentiation during expansion in monolayer culture of autologous chondrocytes represents the main limitation in tissue engineering and cartilage repair; it is reasonable to propose the use of MCh so as to overcome this obstacle. Additionally, results herein presented suggested the possible use of MCh hydrogel as an injectable cell delivery system for cell-based cartilage repair in preclinical studies as well as its possible application in clinical settings.

## Figures and Tables

**Table 1:** Oligonucleotide primers used in this study.

Primers	Sequences (5′–3′)	Accession Number
*GAPDH*	ATCTCGCTCCTGGAAGATG ^a^ TCGGAGTGAACGGATTCG ^b^	NM_001034034
*18S*	TTCGATGGTAGTCGCTGTGC ^a^ TTGGATGTGGTAGCCGTTTC ^b^	NR 036642
*Acan*	CATCCCCTGCTACTTCATCG ^a^ CCTTCTCCTTGGAAATGCG ^b^	NM_173981
*Col1A2*	GGATGGTCACCCTGGAAAAC ^a^ CCCCTAATGCCCTTGAAGC ^b^	NM_174520
*Col2A1*	TGATCGTGGTGACAAAGGTG ^a^ ATCTGGGCAGCAAAGTTTCC ^b^	NM_001001135
*Sox9*	ACGCAGATTCCCAAGACAC ^a^ GGTTTCCAGTCCAGTTTCG ^b^	XM_014478986

^a^ Forward primer; ^b^ reverse primer.

## Supplementary Materials

Supplementary materials can be found at https://www.mdpi.com/1422-0067/21/16/5798/s1.
