# Supplementary material for: Production of Injectable Marine Collagen-Based Hydrogel for the Maintenance of Differentiated Chondrocytes in Tissue Engineering Applications
Source: Int J Mol Sci. 2020 Aug 12;21(16):5798. doi: 10.3390/ijms21165798 (PMC7461064; doi:10.3390/ijms21165798)
Supplement: Supplementary file 1 [file ijms-21-05798-s001.pdf]

International Journal of
Molecular Sciences

MDPI

Supplementary Material

Table S1. After the desalination process, each sample was weighed and from this value it was possible to obtain both the amount of water, which represents approximately 98% by weight, and the hypothetical amount of proteins, mainly collagen, for the remaining 2% in weight.

	**Fresh Weight (gr)**	**Amount of Water (mL) ≈ 98%**	**Amount of Proteins (gr) ≈ 2%**	**Protein Concentrations (mg/mL)**
Umbrella 1#	560	557.62	11.38	20.41
Oral Arms 1#	604.17	592.09	12.08	20.4
Umbrella 2#	411.6	403.37	8.23	20.4
Oral Arms 2#	214.48	210.19	4.29	20.41

Table S2. Respective volumes of water and acetic acid [17,5 N] to added at each sample to obtain a final concentration solution of: [0,05 N] (**A**); [0,1 N] (**B**);[0,5 N] (**C**).

(**A**)

	**Amount of Water into the Sample (mL)**	**Complementary Quantity of Water to Obtain a Final Volume of 600mL**	**mL of CH3COOH [17.5 N] Added to the Final Volume**	**(CH3COOH) Cf for 6 Days**
Umbrella 1#	557.62	40.66	1.72	0.05 N
Oral Arms 1#	592.09	6.19	1.72	0.05 N
Umbrella 2#	403.37	194.91	1.72	0.05 N
Oral Arms 2#	210.19	388.09	1.72	0.05 N

(**B**)

**Volume of Each Solution**	**Increase in CH3COOH [17.5 N] Concentration (mL Added to Each Sample)**	**(CH3COOH) Cf for 10 Days**
600 mL	1.71 mL	0.1 N

(**C**)

**Volume of Each Solution**	**Complementary Quantity of Water to Obtain a Final Volume of 630mL**	**Increase in CH3COOH [17.5N] Concentration (mL Added toEach Sample)**	**(CH3COOH) Cf for 4 Days**
601.71 mL	6.86 mL	18 mL	0.5 N

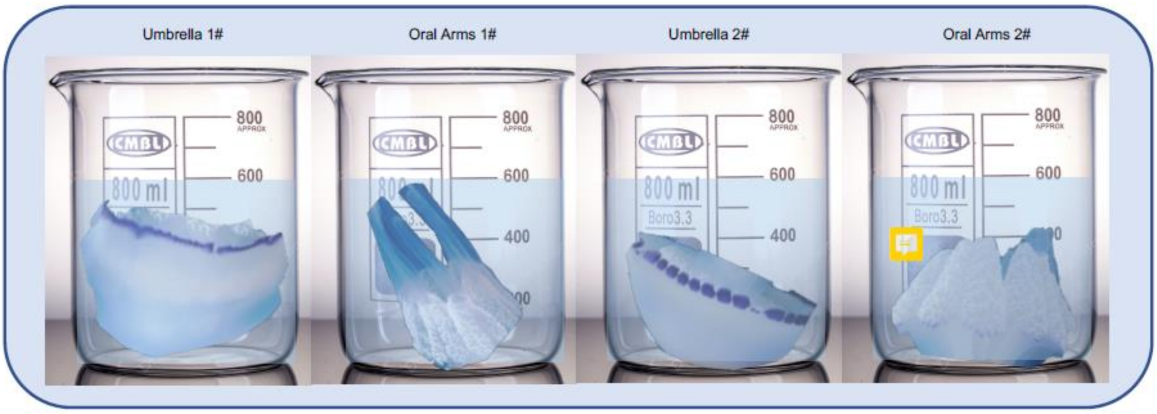

Figure S1. Each sample was incubated in a gradually increasing concentration of acetic acid solution up to the concentration of [0.5 N]. The protein extraction process was carried out at 4 °C for a total of 20 days.
